# The significance of the Van Nuys prognostic index in the management of ductal carcinoma *in situ*

**DOI:** 10.1186/1477-7819-6-61

**Published:** 2008-06-18

**Authors:** Onur Gilleard, Andrew Goodman, Martin Cooper, Mary Davies, Julie Dunn

**Affiliations:** 1 The Royal Devon and Exeter Breast Cancer Unit, Exeter, Devon, EX2 5DW, UK

## Abstract

**Background:**

Debate regarding the benefit of radiotherapy after local excision of ductal carcinoma *in situ *(DCIS) continues. The Van Nuys Prognostic Index (VNPI) is thought to be a useful aid in deciding which patients are at increased risk of local recurrence and who may benefit from adjuvant radiotherapy (RT). Recently published interim data from the Sloane project has showed that the VNPI score did significantly affect the chances of getting planned radiotherapy in the UK, suggesting that British clinicians may already be using this scoring system to assist in decision making. This paper independently assesses the prognostic validity of the VNPI in a British population.

**Patients and methods:**

A retrospective review was conducted of all patients (n = 215) who underwent breast conserving surgery for DCIS at a single institution between 1997 – 2006. No patients included in the study received additional radiotherapy or hormonal treatment. Kaplan Meier survival curves were calculated, to determine disease free survival, for the total sample and a series of univariate analyses were performed to examine the value of various prognostic factors including the VNPI. The log-rank test was used to determine statistical significance of differential survival rates. Multivariate Cox regression analysis was performed to analyze the significance of the individual components of the VNPI. All analyses were conducted using SPSS software, version 14.5.

**Results:**

The mean follow-up period was 53 months (range 12–97, SD19.9). Ninety five tumours were high grade (44%) and 84 tumours exhibited comedo necrosis (39%). The closest mean initial excision margin was 2.4 mm (range 0–22 mm, standard deviation 2.8) and a total of 72 tumours (33%) underwent further re-excision. The observed and the actuarial 8 year disease-free survival rates in this study were 91% and 83% respectively. The VNPI score and the presence of comedo necrosis were the only statistically significant prognostic indicators (P < 0.05).

**Conclusion:**

This follow-up study of 215 patients with DCIS treated with local excision and observation alone is one of the largest series in which rates of recurrence are unaffected by radiation therapy, hormone manipulation or chemotherapy. It has afforded us the opportunity to assess the prognostic impact of patient and tumour characteristics free of any potentially confounding treatment related influences. The results suggest that the VNPI can be used to identify a subset of patients who are at risk of local recurrence and who may potentially benefit from RT.

## Background

Screening mammography has led to a significant increase in the reported incidence of ductal carcinoma *in situ *(DCIS) in the last 2 decades and it currently makes up approximately one fifth of all newly diagnosed breast cancers [1]. Whilst many agree that local excision is the preferred treatment for DCIS the debate regarding the use of adjuvant radiotherapy (RT) after such surgery is currently one of the most controversial areas in breast cancer management [2,3]. Findings from 3 independent phase III trials [4-6] have demonstrated that RT reduces the risk of local recurrence by approximately 50%. Limitations in the methodology of these studies, such as failure to routinely measure margins, and the observation that RT does not seem to influence overall survival has led to a lack of consensus regarding its utility [2].

Recently published results from a multi-centre audit, conducted in the UK, have demonstrated a lack of standardization in the use of RT for DCIS across different breast cancer units [7]. Out of 69 participating units, 6 (including our own) withheld it as a primary treatment measure. Two units gave it to all of their patients with DCIS whilst the majority of centres based their decision to give or withhold RT on the presence or absence of certain tumour characteristics thought to influence the likelihood of recurrence. Of these tumour size greater than 15 mm, intermediate or high nuclear grade, presence of comedo necrosis and intermediate or high VNPI scores were found to significantly increase the chance of patients receiving adjuvant RT.

The VNPI itself is a simple scoring method that has been used in the US for some 10 years to stratify patients with different risks of local recurrence although recently its validity has been questioned [8]. The index is based upon grade, size, presence or absence of comedo necrosis and margin width (Table 1) [9]. Results from a recent retrospective study [10] on the influence of patients' age, has led to a modification of the VNPI using age as an additional fourth parameter in the scoring system.

**Table 1:** Van Nuys Prognostic Index

**Predictor**	**Score**		
	1	2	3

Size of tumour (mm)	≤ 15	16–40	>40
Margin width (mm)	>10	1–10	<1
Grade	Non high grade, no comedo necrosis	Non high grade with comedo necrosis	High grade with or without comedo necrosis

In this paper we have applied the original and modified VNPI to prospectively collected data from 215 patients, all of whom were treated with wide local excision alone.

## Patients and methods

Two hundred and fifteen patients underwent breast conserving surgery for DCIS at The Royal Devon and Exeter Hospital between 1997 – 2006. In order for margin width to be determined accurately and in a standardized fashion each specimen had its lateral, medial, cranial, caudal, deep and superficial margins orientated and marked with coloured ink in theatre before being sent for histological analysis. It is our policy to excise all DCIS down to the fascia of pectoralis major and then perform re-excision if the circumferential margins are deemed close (<2 mm). The anatomical constraints obviously limit further excision of close margins in the cross sectional plane and there is no advantage to be gained in re-excision if DCIS approaches the margin adjacent to pectoralis fascia.

All patients were subject to a multi disciplinary review and those with high grade DCIS greater than 1 cm were referred to an oncologist for discussion regarding the potential benefits and side effects of RT. Nine patients treated within this time frame accepted adjuvant RT and as such have been excluded from the study. Patients that were found to have simultaneously occurring invasive disease at the time of diagnosis were excluded from the study as were those who underwent mastectomy, with or without reconstruction, as a primary procedure (n = 135).

All the prospectively entered data regarding patient and tumour characteristics were retrieved from the *dendrite software program *and the following information was recorded: age at diagnosis, nuclear grade, histological pattern, presence or absence of comedo necrosis, size of lesion, closest coronal margin, closest cross-sectional margin, whether re-excision surgery had been performed and if so the presence or absence of disease at the margins. The length of follow-up was recorded together with information on recurrence, presence of metastasis, death and cause of death. VNPI scores were calculated using both the original and modified criteria.

The length of the follow-up period was calculated from the date of the first surgical procedure to the date of the last mammogram or ultrasound. A local recurrence was defined as a pathology-proven carcinoma anywhere in the treated breast including those that occurred in different quadrants to the original tumour. In keeping with similar studies "contralateral recurrences" were not deemed treatment failures.

Kaplan Meier survival curves were calculated for the total sample. The log rank test was used to determine the statistical significance in comparative survival for a variety of patient and tumour characteristics. Cox regression analysis was performed to assess the significance of multiple predictors of disease free survival. All analyses were conducted using SPSS software, version 14.5.

## Results

Table 1 lists the patient and tumour characteristics of the study population. The mean age at diagnosis was 60.3 years (range 33–91, standard deviation 9.3). The mean follow-up period was 53 months (range 12 – 97, standard deviation 19.9). The mean tumour size was 12.2 mm (range 0 – 41, standard deviation 9.9), mean closest margin was 2.4 mm (range 0 – 22, standard deviation 2.8), the number of high grade tumours was 95 (44%) and the number exhibiting comedo necrosis was 84 (39%). In 18 cases (8%) the closest margin width was not specified because, in the early years of the study (1997–1999), when margins were found to be greater than 5 mm the exact width was often not documented and reported only as clear. When reporting the data regarding the influence of margin width (and consequently the VNPI) on disease free survival, we have not included this small minority of tumours in our analysis.

Sixty five patients were found to have margins less than 1 mm on primary excision (Table 2). In 55 of these cases it was found that the circumferential margin was closest and as a result these patients underwent further re-excision. Final margins were greater than 1 mm in all of these cases. A further 17 patients from the group that had initial margins between 1–5 mm underwent further surgery resulting in a total re-excision rate of 33% (n = 72).

**Table 2:** Patient and tumour characteristics

**Characteristic**	**N**	**%**
**Age at diagnosis**		
<60	104	48
≥ 60	111	52

**Histological subtype**		
Comedo	54	25
Cribriform	49	23
Solid	7	3
Papillary	22	10
Mixed	49	23
Not specified	34	16

**Nuclear grade**		
Low	69	32
Intermediate	51	24
High	95	44

**Comedo necrosis**		
Yes	84	39
No	130	60
Not specified	1	<1

**Tumour size (mm)**		
<5	46	21
5–10	63	29
11–20	55	26
>20	34	16
Not specified	17	8

**Closest margin (mm)**		
<1	65	30
1–5	102	47
>5	30	15
Not specified	18	8

**Re-excision**		
Yes	72	33
No	143	67

**VNPI**		
3–4	61	29
5–7	104	48
8–9	20	9
Not specified	30	14

**Figure 1:**
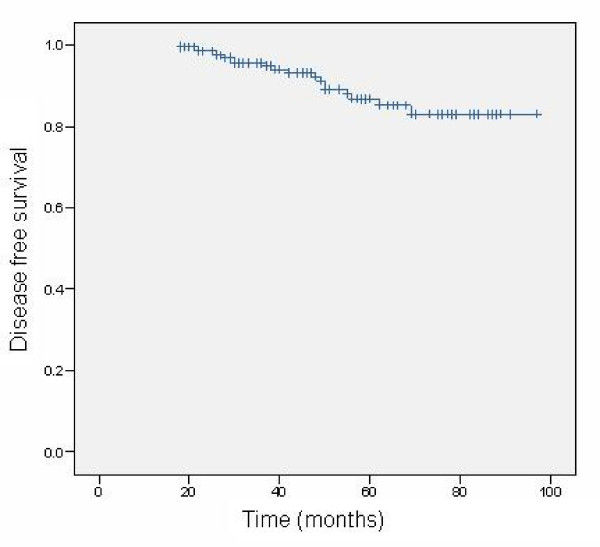
Predicted 8 year disease free survival curve.

There were 8 non invasive and 11 invasive recurrences in the treated breast during the follow up period. The estimated 8 year disease free survival was 83% (Table 3 and Figure 1). Mean time from surgery to any recurrence was 32.1 months. There were 2 breast cancer related deaths. One occurred in a patient who developed contralateral invasive breast cancer and the other in a patient who developed invasive disease in the treated breast. Additionally 1 patient died from metastatic colorectal adenocarcinoma.

**Table 3:** Eight-year local recurrence free survival calculated using the Kaplan-Meier method

**Event**	**N**	**8-year recurrence free survival (%)**
All recurrences	19	83
Invasive	11	87

**Figure 2:**
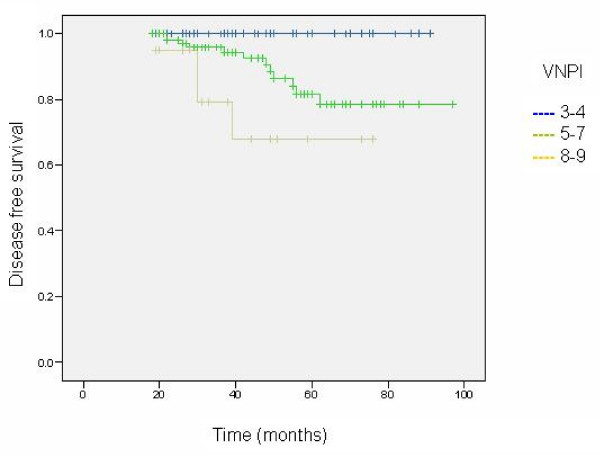
The influence of the VNPI on disease free survival.

Table 4 shows estimated 8 year disease free survival for selected patient and treatment characteristics. The VNPI and the presence of comedo necrosis were the only factors to significantly influence outcome (Table 4 and Figure 2). In this study age did not significantly affect outcome and as a result the modified VNPI was not found to be a predictor of recurrence.

**Table 4:** Predicted 8-year local recurrence free survival for selected patient and treatment characteristics

**Characteristic**	**Predicted 8 year local recurrence free survival (%)**	**P value**
**Age**		
<60	83.0	0.68
≥ 60	82.7	

**Re excision**		
Yes	80.4	0.48
No	84.7	

**Nuclear grade & comedo necrosis**		
Non high grade, no comedo necrosis	89.9	
Non high grade with comedo necrosis	82.7	0.04
High grade with or without comedo necrosis	73.8	

**Tumour size (mm)**		
≤ 15	91.0	
16–40	80.2	0.42
>40	100	

**Closest margin (mm)**		
<1	75.8	0.17
1–10	86.5	
>10	97.2	

**VNPI**		
3–4	100	
5–7	78.5	0.002
8–9	67.9	

## Discussion

In contrast to the well established prognostic factors determining outcome in invasive breast carcinoma [11], the value of similar prognostic indices has proved less clear cut in DCIS. The present study of 215 patients with DCIS treated with local excision and observation alone is one of the largest series in which recurrence is unaffected by radiation therapy, hormone manipulation or chemotherapy and has given us the opportunity to assess the prognostic impact of patient and tumour characteristics free of any potentially confounding treatment related influences.

In this study we have shown that for those patients with a low VNPI score (scores 3–4, n = 61) the recurrence rate and hence the chance of developing invasive breast cancer is minimal (0% over 8 years, P = 0.002). These patients we feel should not receive RT. For those with intermediate (scores 5–7, n = 104) and high (scores 8–9, n = 20) VNPI scores the chance of developing any recurrence over 8 years in this study is 21.5% and 32.1% respectively (P = 0.002). Taking these factors in to account and appreciating that the natural history of DCIS remains elusive, it is our opinion that RT should be reserved for those patients with high and possibly intermediate VNPI scores as it is in these groups that the benefit: risk ratio is likely to be highest.

The effect of including the small number of patients with tumours that did not have their margin width recorded (n = 18) in the analysis of the VNPI's effect on disease free survival would re-enforce its significance, as all had low scores (3–4) and in none of the cases was a recurrence observed.

Comedo necrosis was found to be present in 84 cases (39%) and when analysed in combination with grade of tumour, as specified in the VNPI, was found by univariate analysis to adversely influence disease free survival (p < 0.05). In Cox multivariate regression analysis, none of the individual components of the VNPI reached statistical significance, suggesting that the whole Index is of greater value than its parts. Adding age to the index reduced rather than increased its prognostic value.

Obviously it is important to note that the retrospective nature of this study means that conclusions must be drawn with caution. There is currently a wealth of relatively small series of studies and personal opinions regarding the decision to give or withhold RT as a primary treatment measure in DCIS [2,3,12,13]. Results and opinions are often conflicting. Advocates for giving this modality point to the fact that the only level I evidence that is available, the gold standard in today's evidence-based practice, demonstrates without question that RT reduces local recurrence [4-6]. Furthermore it has been suggested that the reason why a survival benefit has not been demonstrated in the large randomised trials is due simply to the fact that the follow up period has not yet been long enough [3].

In contrast there are clinicians on both sides of the Atlantic who feel the methodology of the aforementioned trials, especially regarding the measurement of margin width which has been shown by certain authors to be a determinant of local recurrence [14], raises concerns about the significance and therefore applicability of the results. Those who are reluctant to use RT for DCIS as a primary treatment argue that a substantial proportion of lesions behave in a benign fashion and are unlikely to transform into carcinoma during the patient's life-time [15] and as such it is unreasonable to indiscriminately subject the increasingly large number of women with screen detected DCIS to the potentially serious side effects of RT, when such therapy has yet to demonstrate a survival benefit.

Perhaps the most convincing evidence against adopting such a stance has been described by Wong *et al*., [16]. These authors conducted a single arm prospective trial evaluating recurrence rates after breast conserving surgery alone in a group of patients in which they predicted that the rate of recurrence would be low (margins >1 cm, low/intermediate grade DCIS). The trial was prematurely stopped after the predefined boundaries for what was deemed as an acceptable recurrence rate was overstepped. The estimated 5 year ipsilateral local recurrence rate in the 158 patients accrued was 12%, which is a value similar to the surgery only arms of the UKCCCR, EORTC and NSABP trials [4-6] and as such appeared to support the conclusion that there is in fact not a subgroup of patients with DCIS, for whom RT should not be offered.

Silverstein and Lagios [2] have highlighted various factors in the methodology of this study which may partially be responsible for the relatively high recurrence rates observed. They also point out that the majority of cases of recurrence were non invasive (69%) in nature and could be treated by re-excision plus or minus RT with an expected 100% cause specific survival. They further calculate that taking into account the cases of invasive recurrence (31%) the expected cause specific mortality at 12 years would be only 0.6% and consequently the harm avoided by withholding RT in 158 patients should result in this trial being viewed not as a failure but rather as a success.

More recently Macausland *et al*., [8] made an attempt to validate the VNPI but found that although trends were observed between this stratification system and local recurrence, none reached statistical significance. A significant number of patients in this cohort received tamoxifen as adjuvant therapy however and this may have influenced results. Additionally the authors acknowledge that the predictive utility of the VNPI in this study may well be seen with further follow-up.

As a consequence of the controversy surrounding the decision whether to give or withhold RT, there is a substantial lack of standardization in the treatment for DCIS at both national and international level [17]. It seems that until there is sufficient level I evidence determining that a certain subgroup of patients who, following wide local excision alone, are shown to have a rate of recurrence that is less than or at least equal to those described in the surgery plus RT arms of the large trials a lack of uniformity will persist. Whether identification of such a subgroup, if it does indeed exist, is to be made using a relatively simple scoring system such as the VNPI, or by the detection of more advanced biological markers is not yet clear [18].

## Conclusion

As the incidence of DCIS continues to rise, particularly in asymptomatic women of screening age, accurately predicting the risk of progression and recurrence is of paramount importance for the formulation of rational treatment strategies [19]. In several British centres, clinicians are using the VNPI to determine whether patients receive adjuvant RT [7]. In this study we have shown that the VNPI is a statistically significant determinant of local recurrence when local excision is the only treatment modality applied. As such its use in determining which patients are most likely to benefit from adjuvant radiotherapy appears to be of value, although further research is needed by way of randomised control trials to determine more precisely the risk: benefit ratio of such a course of action.

## Competing interests

The authors declare that they have no competing interests.

## Authors' contributions

OG participated in data acquisition and interpretation and wrote the manuscript, MD helped in data acquisition, JD and MC carried out the surgical procedures and critically reviewed the manuscript, AG critically reviewed the manuscript. All authors read and approved the manuscript.
